# Treatment of bronchial anastomotic fistula using autologous platelet-rich plasma post lung transplantation

**DOI:** 10.1186/s13019-022-01965-w

**Published:** 2022-08-24

**Authors:** Aisha Siddique, Belal Nedal Sabbah, Tarek Arabi, Ismail Mohammed Shakir, Rayid Abdulqawi, Khaled AlKattan, Mohamed Hussein Ahmed

**Affiliations:** 1 College of Medicine, Alfaisal University, Riyadh, Saudi Arabia; 2 Department of Anatomy and Cell Biology, College of Medicine, Alfaisal University, Riyadh, Saudi Arabia; 3 Lung Health Centre, Organ Transplant Centre of Excellence, King Faisal Specialist Hospital and Research Centre, Riyadh, Saudi Arabia; 4 Department of Surgery, College of Medicine, Alfaisal University, Riyadh, Saudi Arabia; 5 Department of Cardiothoracic Surgery, Ain Shams University, Cairo, Egypt

**Keywords:** Bronchial anastomosis, Bronchial dehiscence, Bronchopleural fistula, Lung transplant, Platelet-rich plasma, Airway complications

## Abstract

**Background:**

Bronchial anastomotic dehiscence is considered one of the most catastrophic early airway complications post-transplant. The presence of a partial dehiscence can also cause further complications such as a fistula between the bronchus and the pleural membrane. Platelet-rich plasma (PRP) is known to significantly enhance the healing process and is being used in the treatment of various conditions, however, so far, there are no reports of the use of PRP in the treatment of bronchial anastomotic dehiscence fistula.

**Case presentation:**

We present a 37-year-old male, with non-cystic fibrosis bronchiectasis underwent bilateral lung transplantation. The patient developed partial dehiscence of the right bronchial anastomosis that was complicated by a small bronchopleural fistula. Two bronchoscopic applications of autologous platelet-rich plasma were carried out. Follow-up a few weeks later showed complete closure and healing of the fistula.

**Conclusions:**

This case report suggests that the treatment of post-lung transplant small bronchial anastomotic partial dehiscence fistula with PRP is safe and effective.

## Introduction and literature review

Transplantation of the lungs is associated with significant post-surgical airway complications which contribute to increased morbidity and mortality. Since the first ever lung transplantation, efforts have been made to identify complications associated with transplant and streamline definitions, grading systems, and management aimed at reducing them. In addition, efforts have been directed towards exploring less invasive novel interventions to manage complications. Currently, the reported incidence for airway complications post-lung transplant ranges from 2 to 18% depending on the definition and grading system used [1]. The spectrum of bronchial complications includes bronchial stenosis, partial dehiscence, formation of granulation tissue, bronchopleural fistula (BPF), strictures, and tracheobronchial malacia. These complications may arise at either the anastomotic site or distal airways, with the right side being affected more than the left lung [2].

Bronchial anastomotic partial dehiscence, which is essentially the splitting open of some sutures at the surgical anastomosis site, is considered one of the most catastrophic early airway complications post-transplant, with an incidence rate of 1–10%. Partial dehiscence of the airway most frequently occurs between 1 and 6 weeks post-transplant and may or may not be accompanied by mucosal necrosis. Partial anastomosis dehiscence may result in pneumothorax with persistent leakage and infection of the lungs and/or pleura. The usually asymptomatic nature and fatality associated with airway dehiscence has mandated the need for routine bronchoscopic surveillance [1]. Infection at the anastomotic site is one of the causes of delayed or poor healing and contributes to dehiscence. The most common pathogens are *Aspergillus*, *Candida*, *Rhizopus*, and *Mucor* [1, 3]. The outcome of airway dehiscence depends on various factors, including the severity of necrosis, extent of dehiscence, and any accompanying infections.

The presence of a partial airway dehiscence can also cause further complications, one of which is the formation of a fistula between the bronchus and the pleural membrane (i.e., BPF), which manifests as pneumothorax or subcutaneous empyema [4]. Anastomotic fistulas can expose the sterile pleural space to endobronchial bacterial flora. The resultant pleural effusion can leak into the major airways and spread to the peripheral alveolar spaces, which can cause severe aspiration pneumonia and/or empyema, which are potentially fatal.

Although there are a wide variety of treatment options available for anastomotic fistulas with varying therapeutic success, there is a lack of consensus regarding the ideal treatment option.

This report presents, for the first time, the successful treatment of a partial bronchial anastomosis dehiscence complicated by BPF in a bilateral lung transplant patient with autologous platelet-rich plasma (PRP). PRP consists of plasma with platelets and growth factors at concentrations much higher than those typically present in blood and is known to accelerate healing and regeneration.

## Case description

### Case presentation

**Fig. 1:**
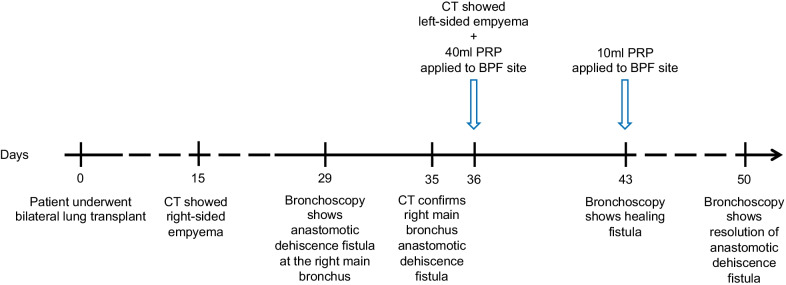
A graphic representation of the timeline of the case. The blue arrows depict the time of PRP application

A 37-year-old male underwent bilateral lung transplantation for non-cystic fibrosis bronchiectasis. The patient developed partial dehiscence of the right bronchial anastomosis complicated by a BPF 29 days post-transplant. Upon assessment there was no evidence of pneumothorax, however, the presence of a small fistula was identified and confirmed both by bronchoscopy and CT imaging. The fistula was found to cause empyema in the right lung. Two bronchoscopic applications of autologous platelet-rich plasma of 40 mL and 10 mL were carried out on post-op days 36 and 43, respectively. Follow-up showed complete closure and healing of the fistula on post-op day 50 (Fig. 1).

### Post-transplant infectious events and complications

The patient underwent a double lung transplant while on central ECMO support and was mechanically ventilated for 2 days. The ischemia times were 5 h 22 min and 7 h 14 min for the right and left lung, respectively. The patient was placed on methylprednisolone, mycophenolate, and tacrolimus for immunosuppression. mTOR inhibitors were not included in the treatment regimen of this patient.

Six days after extubation, the patient developed hypoxic respiratory failure, so he was intubated again and extubated successfully 2 days later. During his ICU stay, the patient developed right sided loculated pleural effusion and empyema; pleural fluid culture grew *Klebsiella pneumoniae*. In addition, he developed donor-derived pneumonia with multidrug-resistant *Pseudomonas aeruginosa* and a carbapenemase-producing *Klebsiella pneumoniae* OXA-48 gene that was complicated by secondary bacteremia. Initially, he was placed on a broad-spectrum antibiotic with colistin that was later changed to ceftazidime-avibactam for 6 weeks. He was also treated with a course of tobramycin for decolonization of MDR *Pseudomonas aeruginosa*. To control the empyema, a right chest tube was inserted for drainage.

**Fig. 2:**
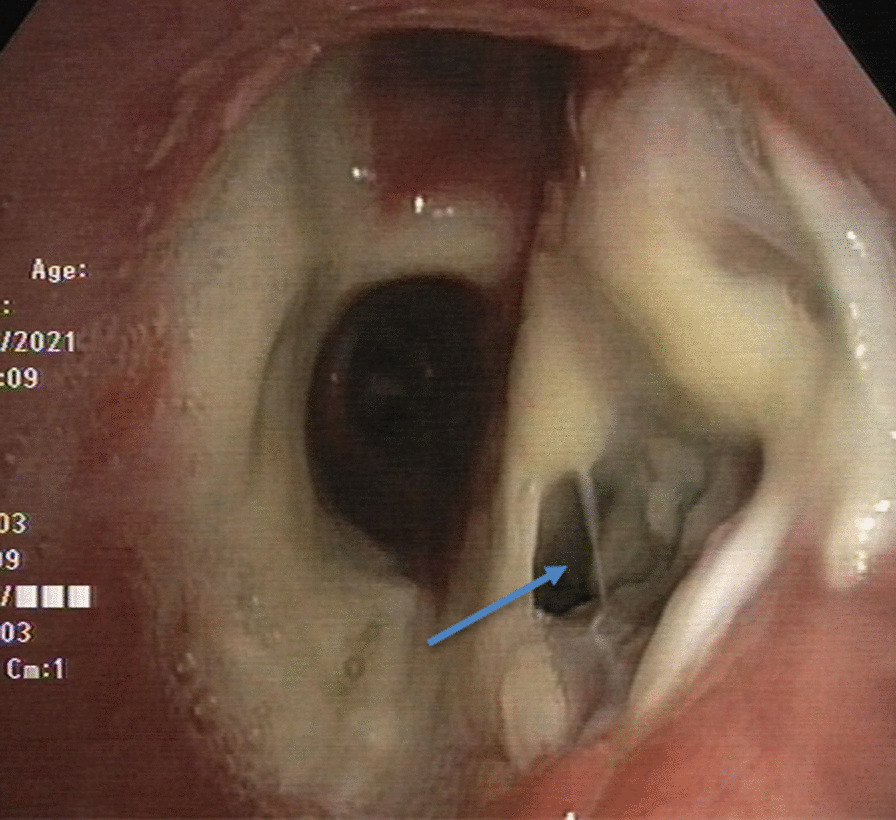
Surveillance bronchoscopy showing the BPF (arrow) at the right main bronchus as the result of partial dehiscence of the anastomosis

**Fig. 3:**
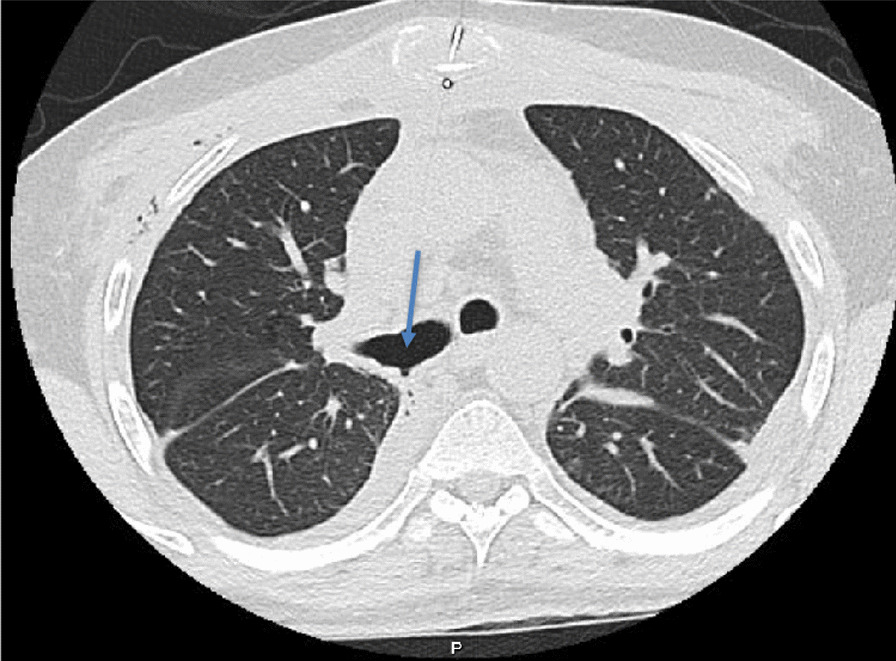
Day 35 post-transplant chest CT showing partial dehiscence and a small bulging contour along the posterior aspect of the right bronchial anastomosis with adjacent tiny air foci (arrow) concerning a partial dehiscence

Surveillance bronchoscopy 1 month post-op showed mucosal sloughing at the areas of anastomosis with possible dehiscence in the right bronchus forming a BPF (Fig. 2). The transbronchial biopsies did not reveal any acute cellular rejection. Chest CT 6 days later showed left-sided empyema with partial dehiscence of the right bronchial anastomosis forming a BPF (Fig. 3). The patient was then scheduled for left-sided chest drain insertion and autologous PRP application at the site of dehiscence using bronchoscopy.

### Preparation of platelet-rich plasma (PRP)

The platelet-rich plasma was prepared after harvesting plasma from a sample of the patient’s blood (whole blood concentration: 238 ± 38 × 10^3^/µL, mean ± SD) using the Arthrex ACP Double Syringe System (ACP, Arthrex concentration: 470 × 10^3^/µL).

### Treatment with platelet-rich plasma

**Fig. 4:**
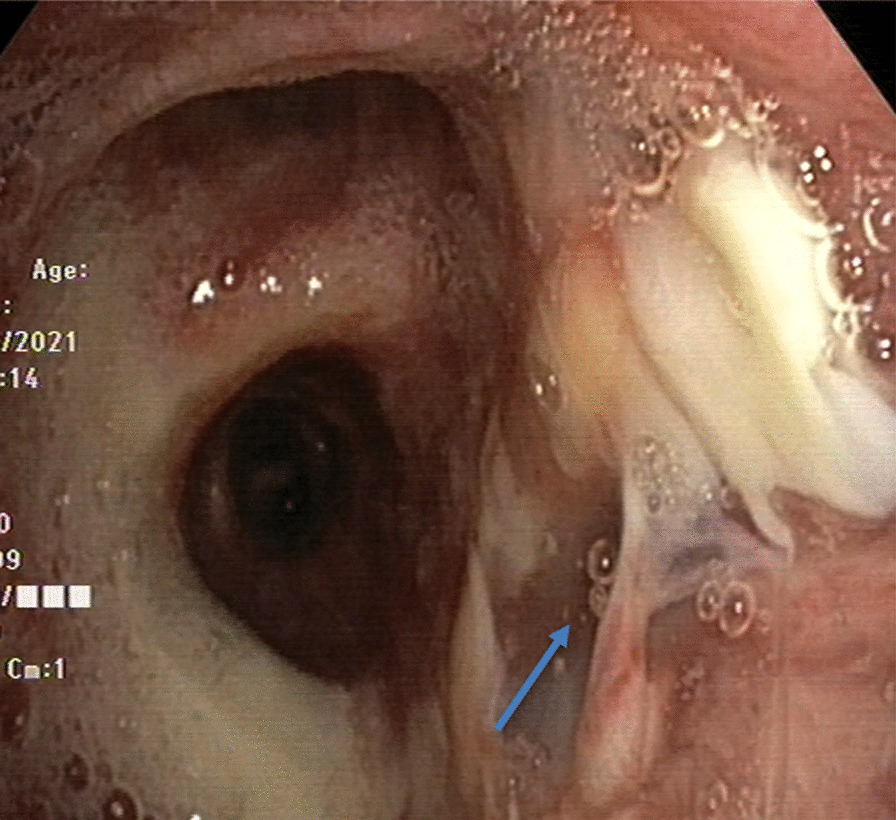
Bronchoscopy on post-op day 50. Previous fistula at the right main stem bronchus completely closed (arrow) after the previous PRP applications

**Fig. 5:**
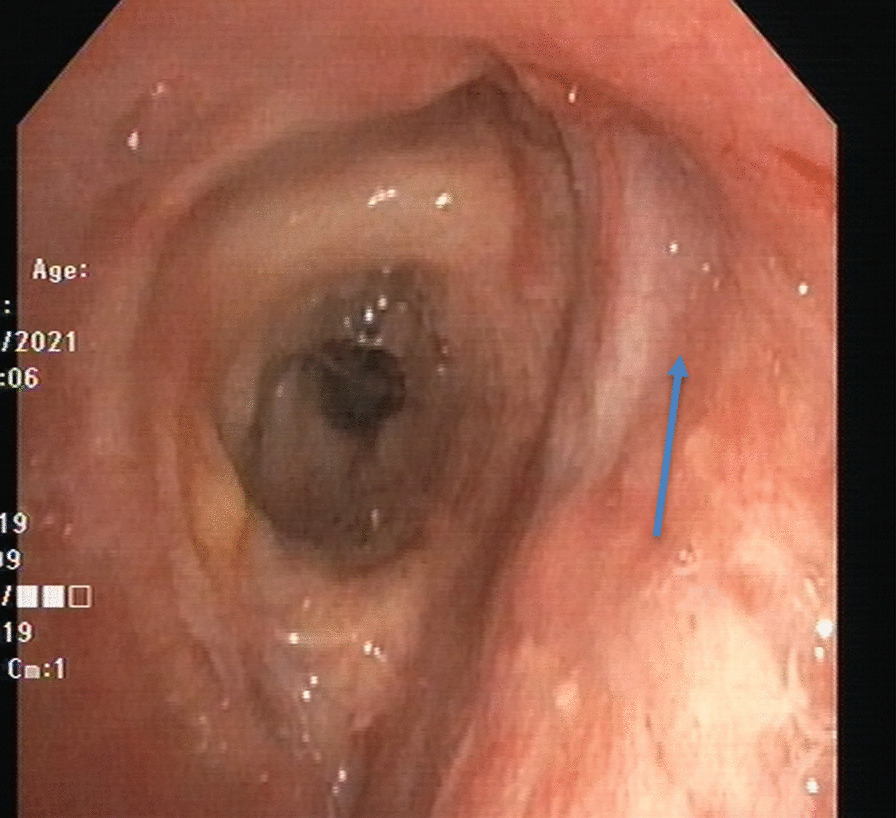
Bronchoscopy on post-op day 162 showing resolution of the fistula (arrow)

Autologous PRP was applied directly via bronchoscopy to the site of the right bronchial dehiscence on two occasions 1 week apart. The first application took place 36 days post-op; 40 mL of PRP was applied to the site of the anastomotic dehiscence fistula via bronchoscopy until completely covered. The second bronchoscopic application of 10 mL was carried out on postoperative day 43, when the old anastomotic fistula was nearly closed. Bronchoscopy performed on postoperative day 50 showed complete closure and healing of the anastomotic fistula, so no further PRP was applied (Fig. 4). Long-term follow-up and surveillance bronchoscopy, postop day 162, revealed resolution of the anastomotic fistula (Fig. 5). Overall, there were no complications, and the patient tolerated all the procedures well.

## Discussion

Post-lung transplantation airway complications contribute significantly to the morbidity and mortality of patients, with ischemia of the donor bronchi being the primary cause of such complications [1]. Bronchi obtain their blood supply from both the bronchial and pulmonary circulations; therefore, they are supplied by well-oxygenated blood from the aorta and/or intercostal arteries, whereas the pulmonary artery circulation contains deoxygenated blood. In lung transplantation, donor bronchial vessels are not connected to the recipient bronchial vessels, leaving the donor bronchi dependent on the low oxygenated pulmonary arterial supply. Vascularization of donor bronchi by the recipient bronchial circulation occurs 2–6 weeks post-transplant. Therefore, low perfusion of the pulmonary circulation in the first 6 weeks may result in airway complications. The main risk factors contributing to airway complications are poor lung preservation, prolonged ischemia times, long donor bronchus, certain surgical techniques, primary graft dysfunction, acute rejection, infection, hypotension, donor-recipient height mismatch, mTOR inhibitors, and prolonged mechanical ventilation/high PEEP [1].

In the management of a partial bronchial dehiscence, deployment of airway stents, both covered and uncovered, has been used through flexible or rigid bronchoscopy. However, their use remains controversial due to the associated risks of infection, migration and bronchial injury. The use of fibrin glue through bronchoscopy has been described in cases of partial dehiscence. Surgical repair of the anastomosis is reserved for severe cases or failure to improve cases. In addition, management includes treating associated complications such as pneumothorax and pleural infection.

In the case presented here, the development of a partial anastomotic dehiscence and subsequent BPF 29 days (intermediate-onset) post-bilateral lung transplant could have been due to predisposition to any of the risk factors, including prolonged use of mechanical ventilation, prolonged hospital stay, donor-derived pneumonia with multidrug-resistant *Pseudomonas aeruginosa*, carbapenemase-producing *Klebsiella pneumonia,* and secondary bacteremia. A recent study showed that carbapenemase-producing *Klebsiella pneumonia* significantly increased the risk of developing bronchial anastomotic dehiscence in post-lung transplant patients [3].

Platelet-rich plasma (PRP) is an autologous serum preparation that contains a high level of platelets [5]. PRP is easily obtainable and has been reported as an effective tool to promote wound healing. This wound healing property is explained by the fact that PRP releases abundant growth factors after application to tissues [6] and enhances healing by increasing the production of collagen and fibroblasts [7]. PRP can be easily prepared, and the storage cost is minimal; however, the potential for clinical use is limited to within 5–8 days of preparation. The use of PRP is steadily increasing and has been employed to treat various pathologies, including but not limited to, perianal fistulas in Crohn’s disease, recurrent vesicovaginal fistulas, oro-antral fistulas, osteoarthritis and various musculoskeletal pathologies [8, 9]. One recent study reported 3 successful cases of autologous PRP injections to resolve one tracheobronchial fistula and two BPFs varying in size (4–8 mm), all post-lobectomies following different pathologies other than post-lung transplant [10] Another study conducted on PRP usage as adjuvant therapy for the resolution of vesicovaginal fistulas reported resolution of the fistula with no complications or adverse effects [11]. Additionally, PRP treatment has been studied in porcine models with tracheal resection, where healing of end-to-end anastomosis was accelerated by promoting the release of platelet-derived growth factors (PDGF) and stimulating trans-anastomotic vessel formation [12].

To the authors’ knowledge, there have been no previous case reports describing the use of PRP in the management of anastomosis fistula post-lung transplant. The innovative application of platelet-rich plasma in the treatment of anastomotic fistulas, particularly in patients with challenging presentations, seems to be an intervention with favorable surgical outcomes. Moreover, the technique discussed in this report may be effective, relatively safe, reproducible, and requires minimal resources at least for small bronchial anastomotic dehiscence fistulas. However, our results are only suggestive and larger studies are required to confirm the safety and efficacy of PRP in the healing of bronchial anastomotic fistulas of larger sizes. Additional studies comparing traditional management methods with PRP treatment for bronchial anastomotic dehiscence fistula are also warranted. In addition, the optimal number, quantity, method, and timing of PRP applications or injections in the context of favorable surgical outcomes must also be determined.

## Data Availability

Not applicable.
